# Purification of a heterodimeric Reelin construct to investigate binding stoichiometry

**DOI:** 10.1007/s00249-020-01465-6

**Published:** 2020-10-14

**Authors:** Liam S. Turk, Daniel Mitchell, Davide Comoletti

**Affiliations:** 1 Child Health Institute of New Jersey, New Brunswick, NJ 08901 USA; 2 Department of Neuroscience and Cell Biology, Robert Wood Johnson Medical School, Rutgers, The State University of New Jersey, New Brunswick, NJ 08901 USA; 3 School of Biological Sciences, Victoria University of Wellington, Wellington, 6012 New Zealand

**Keywords:** Protein purification, Heterodimer, Reelin, Affinity chromatography, FLAG tag, His6 tag

## Abstract

Reelin is a secreted glycoprotein that is integral in neocortex development and synaptic function. Reelin exists as a homodimer with two chains linked by a disulfide bond at cysteine 2101, a feature that is vital to the protein’s function. This is highlighted by the fact that only dimeric Reelin can elicit efficient, canonical signaling, even though a mutated (C2101A) monomeric construct of Reelin retains the capacity to bind to its receptors. Receptor clustering has been shown to be important in the signaling pathway, however direct evidence regarding the stoichiometry of Reelin-receptor binding interaction is lacking. Here we describe the construction and purification of a heterodimeric Reelin construct to investigate the stoichiometry of Reelin-receptor binding and how it affects Reelin pathway signaling. We have devised different strategies and have finalized a protocol to produce a heterodimer of Reelin’s central fragment using differential tagging and tandem affinity chromatography, such that chain A is wild type in amino acid sequence whereas chain B includes a receptor binding site mutation (K2467A). We also validate that the heterodimer is capable of binding to the extracellular domain of one of Reelin’s known receptors, calculating the K_D_ of the interaction. This heterodimeric construct will enable us to understand in greater detail the mechanism by which Reelin interacts with its known receptors and initiates pathway signaling.

## Introduction

Reelin is a large glycoprotein that is secreted as a dimer of almost 800 kDa (D'Arcangelo et al. 1997; Kubo et al. 2002); C2101 forms an intermolecular disulfide bond in the oxidative, extracellular environment (Yasui et al. 2011). Reelin’s binding to the very-low density lipoprotein receptor (VLDLR) and the apolipoprotein E receptor 2 (ApoER2/LRP8) is crucial to proper brain development, specifically the discrete lamination observed in the neocortex, and Reelin’s central fragment (Reelin repeats 3–6) is necessary and sufficient to induce neuronal migration during early development (Jossin et al. 2004). Not only is Reelin essential in the context of neuronal migration and layer formation in the neocortex, but it also has physiological roles in dendrite and spine development in the early postnatal brain and synapse function in the adult brain (Lee and D’Arcangelo 2016). It has furthermore been shown that full length Reelin activates a non-canonical signaling pathway that is independent of the receptors, ApoER2 and VLDLR (Lee et al. 2014). However, in the context of this paper, we focus on the canonical signaling pathway that is mediated through VLDLR and ApoER2 and is responsible for the majority of Reelin’s known roles in brain development and function.

Prior work indicates that to induce canonical, physiological signaling, Reelin must be secreted in its wild-type dimeric form; the Reelin C2101A mutant, which can no longer form an intermolecular disulfide bond, fails to activate the Reelin signaling pathway, as measured by a lack of Dab-1 phosphorylation (Yasui et al. 2011). However, the mutant C2101A can still bind the Reelin receptors, as demonstrated by the crystal structure of a shortened, monomeric Reelin fragment interacting with an extracellular portion of ApoER2 (Yasui et al. 2010).

Necessary for Reelin-receptor binding are residues K2467 and K2360. Mutating either of the lysines to alanines disrupts electrostatic interactions occurring at the binding site with corresponding aspartate and glutamate residues on the receptor that coordinate Ca^2+^ binding, shown to be required for the Reelin-receptor interaction (D’Arcangelo et al. 1999; Yasui et al. 2010). K2467 has been deemed more critical to the protein-receptor interaction as not only does the mutation K2467A completely attenuate binding, but so does the mutation K2467R, evidencing that the core lysine residue is important not only in charge, but also in steric conformation (Yasui et al. 2010).

The reason why wild-type, homodimeric Reelin and its monomeric C2101A mutant can both bind to the protein’s receptors but only dimeric Reelin results in signal activation remains unanswered. Although receptor clustering has been shown to be important in Reelin signaling (Strasser et al. 2004), it remains unknown whether Reelin as a dimer binds to two receptors utilizing the binding site of each Reelin chain to relay its signal or if dimeric Reelin binds its receptor in a conformation inaccessible to its monomeric counterpart. Here we outline the purification of a heterodimeric Reelin construct as a novel tool that will help answer this question. We have established a stable cell-line expressing Reelin’s central, signaling fragment with and without the receptor binding site containing different tags, which we define as heterodimer. Utilizing tandem affinity chromatography, we have purified the heterodimeric product with the aim of utilizing it to understand the importance of stoichiometry in the Reelin signaling pathway using primary neuronal cultures.

## Methods

### Cell culture and transfection

HEK293S cells were grown in Dulbecco’s Modified Eagle’s Medium (DMEM) with 5% v/v fetal bovine serum (FBS), at 37 °C in 5% CO_2_. Stable cell lines were obtained by transfecting HEK293S cells using linear polyethylenimine with the cDNA of the appropriate Reelin construct along with an empty pcDNA3.1 plasmid containing the Geneticin resistant gene to confer G418 resistance. After selection with 800 µg/mL of G418 (Sigma), stable cell lines were maintained in DMEM with 5% v/v FBS and 800 µg/mL of G418.

### Construct design

We co-transfected HEK293 cells with two plasmids encoding differentially tagged constructs of the central Reelin fragment (Reelin repeats 3–6) with and without a receptor binding mutation (K2467A). In particular, one plasmid encoded a FLAG-tagged wild-type construct; the second plasmid contained a His6-tagged K2467A mutant construct. The rest of the constructs are identical and in frame with a human Fc fragment for purification, which can be removed as a HRV-3C protease site is located between the C-terminus of the protein and the start of the Fc domain.

### Protein expression

**Fig. 1:**
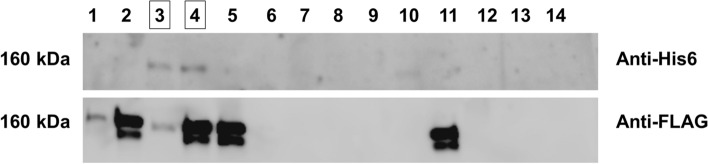
Two clones simultaneously express the differentially tagged protein constructs. SDS-PAGE followed by western blotting of the conditioned media of isolated clones post-selection. Bands were detected at ~ 160 kDa for clones 3 and 4 when probed with antibodies targeting His6 (top) and FLAG (bottom) tags. Two separate gels were ran and blotted independently so there would be no signal carry-over from the previous antibody. Lanes 1, 2, 5, and 11 were not of interest as they only expressed the FLAG-tagged protein, but not the His6-tagged protein. All lanes are shown to provide a sense for the low likelihood of the double expression event

After selection of a stable cell line, we isolated a clone expressing both proteins, relying on the established concept that some fraction of the secreted pool of proteins will be heterodimeric, comprised of the WT and K2467A mutant chains. To identify a clone expressing both chains we used western blotting, probing first with an antibody targeting the His6 tag, followed by an antibody targeting the FLAG tag. Of the roughly 50 colonies isolated and tested during the clonal selection, we detected the presence of both protein constructs in the conditioned media of 2 of the colonies (Fig. 1). These clones were subsequently expanded, frozen, and used to produce the secreted heterodimer on a larger scale in Nunc™ TripleFlask™ cell culture flasks, collecting and replenishing the media on a regular basis until we had roughly 4 L of conditioned medium containing the pool of secreted proteins.

### Purification

**Fig. 2:**
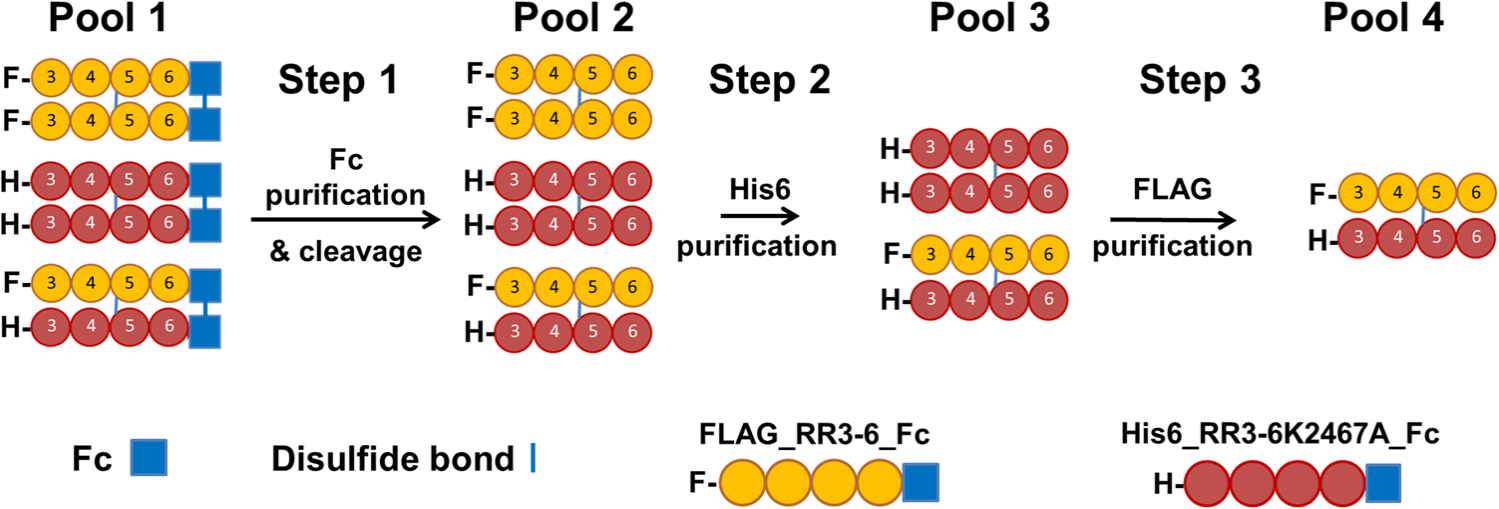
Schematic representation of the tandem affinity chromatography purification strategy utilized to isolate RR3-6 heterodimer. The strategy relies on sequential steps of affinity chromatography, targeting the differentially tagged chains to select for a discrete pool of proteins. Each step gives a new pool of partially purified protein, until pool 4, for which the product is the pure heterodimer

**Fig. 3:**
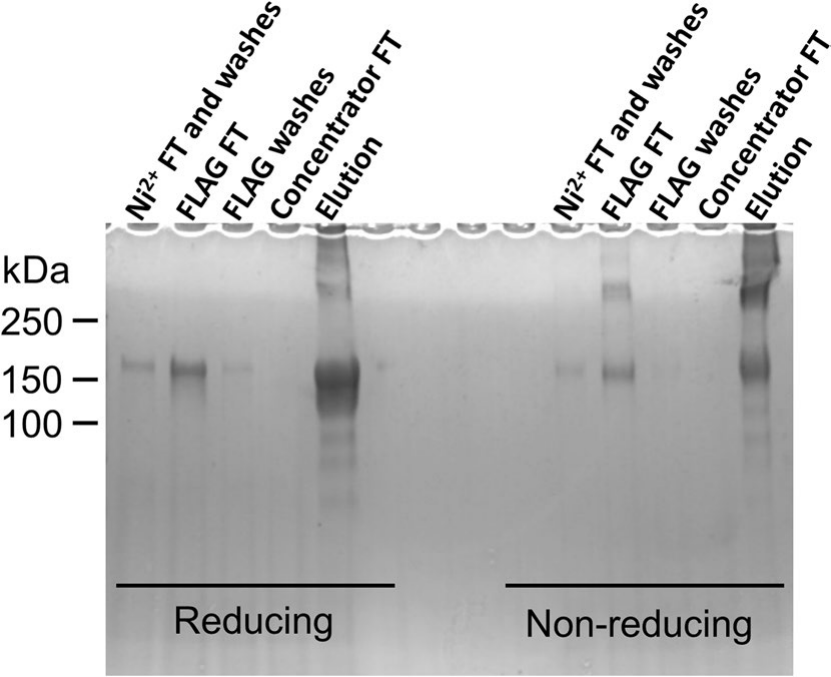
SDS-PAGE characterization of each elution and wash fraction supports the purification strategy. SDS-PAGE followed by Coomassie blue staining of the various stages of purification under reducing and non-reducing conditions. *FT,* flow through; *Ni*^2+^, IMAC Ni-Charged Resin; *FLAG,* anti-FLAG resin. The reduced monomer is expected at ~ 160 kDa. Bands in lanes labeled as FT and washes likely represent homodimeric constructs that are not ultimately purified in the strategy to isolate the heterodimer. The elution lane solely contains the heterodimeric construct

Taking advantage of the fact that, theoretically, a third of the secreted dimeric protein will be comprised of the two different chains, we used tandem affinity chromatography to purify the heterodimer (Fig. 2). Due to our construct design, we first affinity purified all three combinations of homo- and heterodimers using Protein-A Sepharose 4 fast flow resin (pool 1). We then extensively washed the resin (50 mM Tris pH 7.4, 450 mM NaCl), equilibrated it (50 mM Tris pH 7.4, 150 mM NaCl, 1 mM DTT), and eluted the protein (50 mM Tris pH 7.4, 150 mM NaCl, 1 mM DTT, 10 µg/mL HRV-3C protease). The current eluate should contain the two homodimeric FLAG- and His6-tagged constructs as well as the heterodimer (pool 2). The eluate was concentrated to ~ 2 mL using Vivaspin concentrators (Sartorius-Stedim) and further purified using Profinity™ IMAC Ni-charged resin (Bio-Rad) to target the His6-tagged protein chain. We diluted the ~ 2 mL sample with 8 mL of binding buffer (50 mM Tris pH 8, 150 mM NaCl, 5 mM imidazole) and added 2 mL of Profinity resin to the sample. The sample rotated at 4 °C for 30 min, was washed three times with the binding buffer and eluted (50 mM Tris pH 8, 150 mM NaCl, 500 mM imidazole). The resulting eluate (pool 3), contained the His6-tagged homodimer and the His6/FLAG heterodimer. To target the heterodimer in the final purification step, we diluted the ~ 2 mL sample into 8 mL of TN buffer (50 mM Tris pH 8, 150 mM NaCl) and incubated it with 2 mL of Anti-FLAG^®^ M2 Affinity Gel for 2 h at 4 °C while rotating. The saturated resin was then washed three times with TN buffer and eluted with FLAG^®^ peptide (pool 4). These purification steps were followed through the collection of various washes and flow-through which were analyzed using SDS-PAGE on a 4–20% polyacrylamide gel (Bio-Rad) in reducing and non-reducing conditions (Fig. 3). Throughout the experiment and at the final step we obtained a product with the expected MW of 160 kDa in reducing conditions, representing the homodimeric constructs excluded by the affinity chromatography steps, and the heterodimer enriched by the sequential elutions, respectively. The heterodimeric sample was then concentrated to an appropriate volume for validation and downstream experiments.

## Validation

**Fig. 4:**
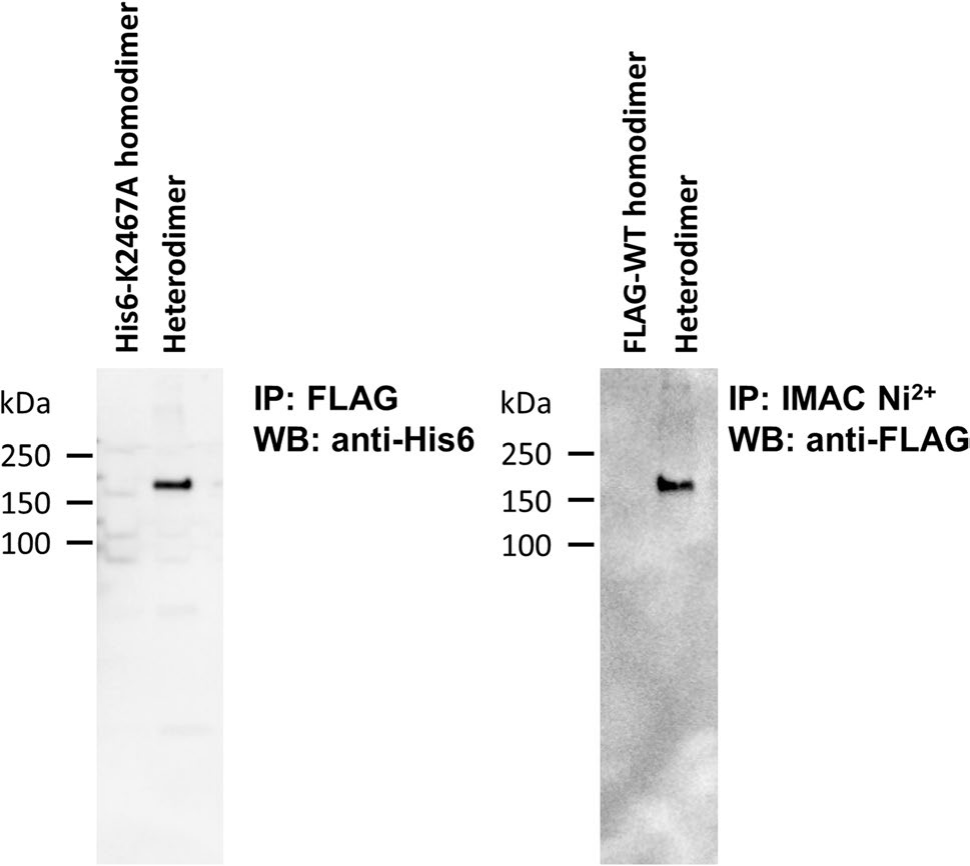
Immunoprecipitation confirms the eluted protein’s heterodimeric composition. Left—FLAG pull down and anti-His6 western blot of His6-K2467A homodimer (lane 1) and FLAG-WT/His6-K2467A heterodimer (lane 2). Right—IMAC Ni^2+^ pull down and anti-FLAG western blot of FLAG-WT homodimer (lane 1) and FLAG-WT/His6-K2467A heterodimer (lane 2). Detection of a band in lanes 2 of each gel supports the heterodimeric composition of the construct, and lack of detection in lanes 1 demonstrates the specificity of the IP in pulling down the targeted protein

To validate the purification and confirm that the construct purified is a heterodimeric construct of the central Reelin fragment, we utilized immunoprecipitation (IP) and western blotting (Fig. 4). Using anti-FLAG resin (Sigma A2220), we pulled down the purported heterodimer and probed for the presence of the His6 tag (Abcam 18184). Detection of a discrete His6-tagged band at 160 kDa, supports the purification strategy and the presence of a heterodimer (Fig. 4, left—lane 2). To confirm that we are not detecting nonspecific binding to the anti-FLAG resin, we incubated the His6-K2467A homodimeric fragment of Reelin (His6-RR36_K2467A) with the anti-FLAG beads and probed for the His6-tag. The absence of any signal argues that the IP was specific for the FLAG-tagged chain (Fig. 4, left—lane 1). To further confirm this, we repeated the experiment in reverse order, performing an IP using IMAC Ni^2+^ resin (Bio-Rad 1560133), then probing for the FLAG tag (Sigma F3165). As expected, we obtained a band at 160 kDa when using the IMAC Ni^2+^ resin to pull down the heterodimer but not when the FLAG-WT homodimer (FLAG-RR36_WT) was incubated with the same resin (Fig. 4, right).

**Fig. 5:**
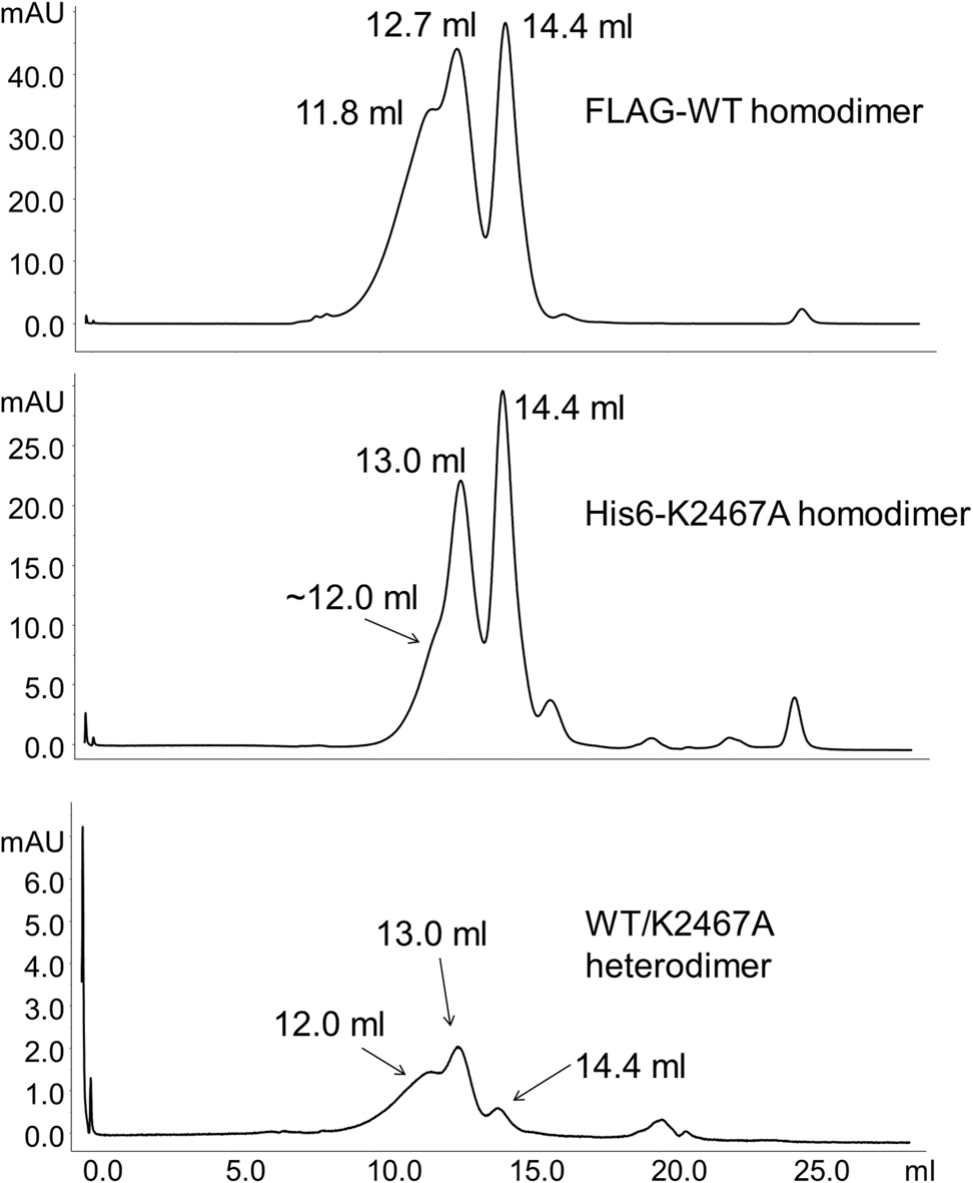
WT/K2467A heterodimer has a similar SEC elution profile to those of the WT and K2467A homodimers. SEC profiles and elution volumes of FLAG-WT homodimer (top), His6-K2467A homodimer (middle), and WT/K2467A heterodimer (bottom). Elution volumes are in mL. 12 mL peak—uncharacterized high order oligomer; 13.0 mL peak—dimer; 14.4 mL peak—monomer

To validate that the protein remains a dimer in solution, we used size exclusion chromatography (SEC). We ran the heterodimeric product of the tandem affinity purification through a Superose^®^6 Increase 10/300 GL column (GE Healthcare) and compared the UVA280 trace to those of the purified FLAG-WT homodimer and His6-K2467A homodimer. The heterodimer elutes with a similar profile to those of the other proteins. There is a left-hand shoulder which is an uncharacterized higher order oligomer (~ 12.0 mL), a main dimeric peak (~ 13 mL), followed by a small peak that is consistent with the monomer (Fig. 5). The small monomeric peak (14.4 mL) visible in the heterodimer trace is likely due to some carry-over during the purification process or the result of a partial reduction of the disulfide bond responsible for maintaining Reelin’s dimeric configuration. However, the ratio of absorbance intensities (monomer:dimer) is greatly reduced when compared with the intensities of the other two constructs for which SEC traces are provided. This is consistent with the selection of a dimeric protein (13 mL peak) during the tandem affinity purification.

**Fig. 6:**
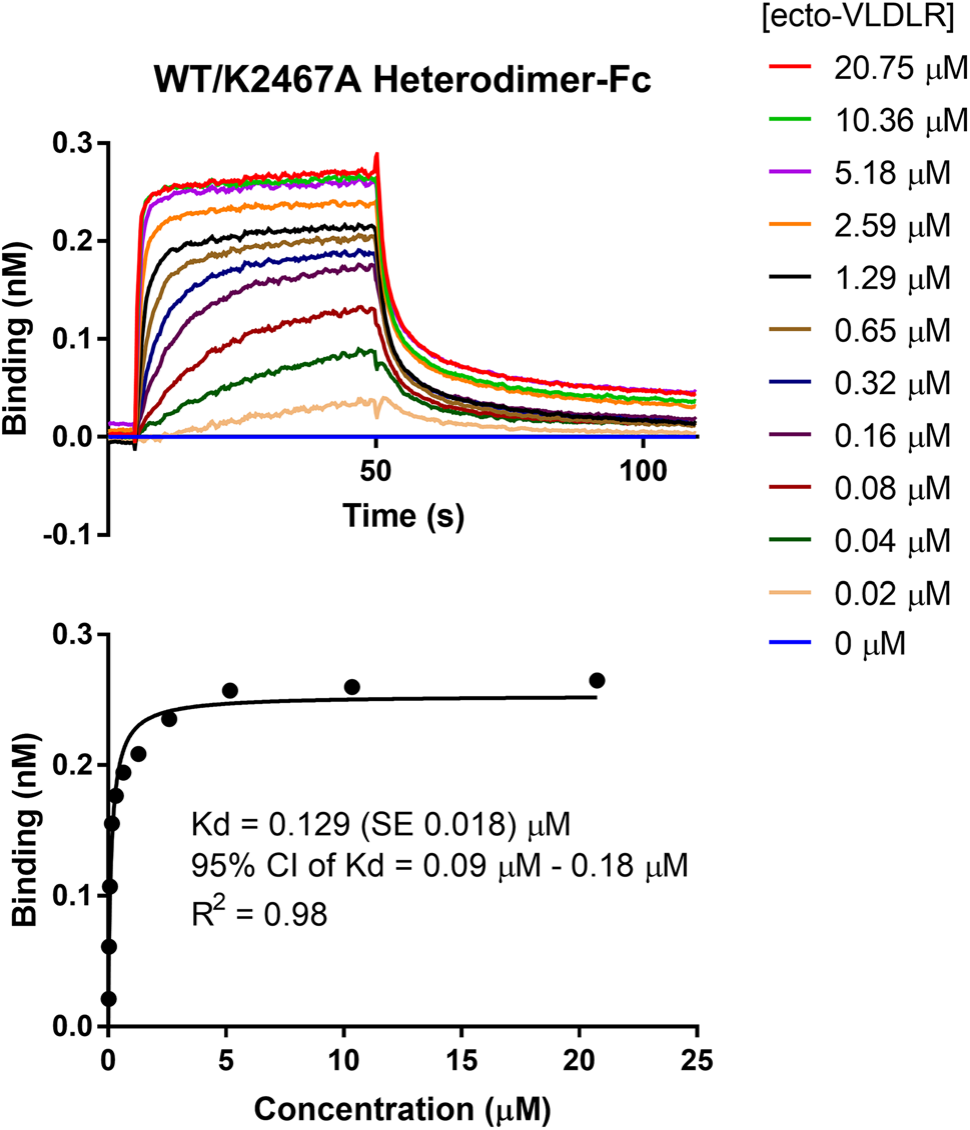
Heterodimeric construct binds to ecto-VLDLR with a *K*_D_ of 129 nM. BLI was used to determine the binding affinity between Fc-fusion Reelin heterodimer and the purified extracellular domain of VLDLR (ecto-VLDLR). The heterodimer was bound to a Protein A biosensor then subject to 11 concentrations of ecto-VLDLR. Association starts at 5 s, and dissociation starts at 50 s. Maximum responses at each concentration (top) were plotted and the data were fitted using a one site—specific binding, nonlinear regression (GraphPad) to determine a *K*_D_ of the interaction as well as the 95% confidence interval (CI, bottom). All curves were done in triplicate and the averaged signals are plotted

**Fig. 7:**
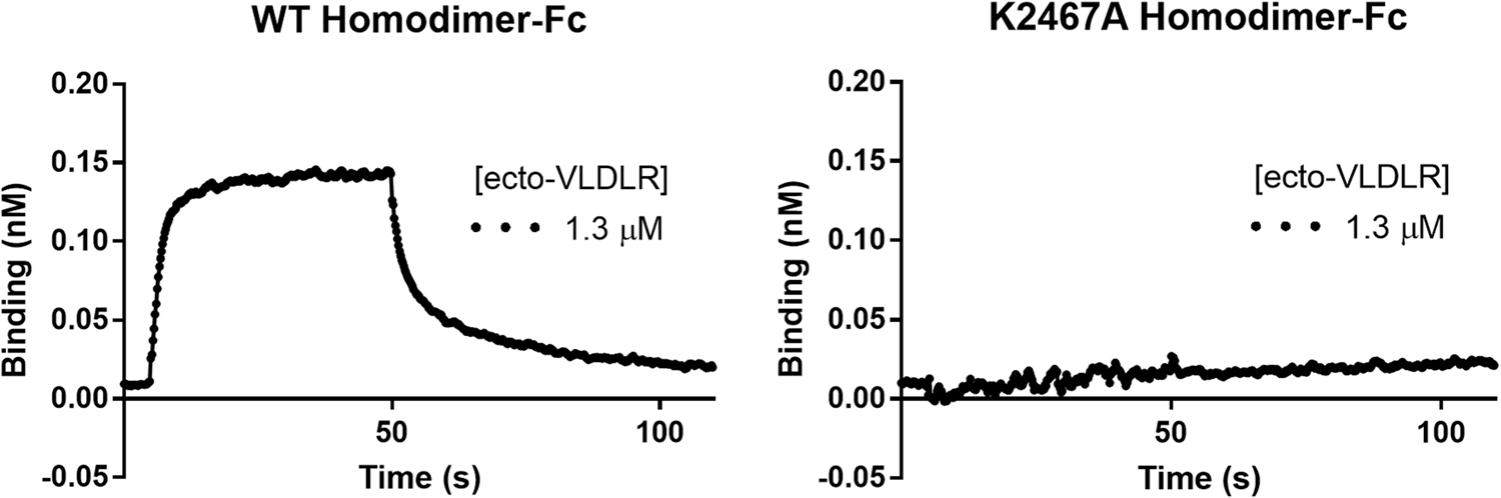
BLI confirms that the WT homodimer interacts with VLDLR, while the K2467A homodimer is inert. BLI experiments were conducted between Reelin-Fc constructs bound to a Protein A biosensor and purified ecto-VLDLR at a concentration of 1.3 µM. Association starts at 5 s, proceeds for 45 s, and is followed by a 60 s dissociation phase. The WT homodimer demonstrates characteristic phases of association followed by dissociation (left) that represent its capacity to bind to ecto-VLDLR. The K2467A homodimer shows no activity (right), as expected due to the disrupted receptor binding site (Yasui et al. 2010)

Lastly, we used bio-layer interferometry (BLItz, FortBio, Menlo Park, CA) to validate that the heterodimer retains, at least in part, the capacity to bind to the purified extracellular domain of VLDLR (ecto-VLDLR), one of Reelin’s known receptors (D'Arcangelo et al. 1999). For this experiment, we purified the heterodimer bypassing the first purification step (Fig. 2), so that our final protein construct retained the Fc moiety. The Fc-fused heterodimer was immobilized to a Protein A biosensor, pre-wetted in 20 mM Hepes pH 7.4, 150 mM NaCl, 1 mM CaCl_2_, 0.2% v/v Tween20, and 0.1% w/v BSA. The BLI experiment proceeded with association for 45 s and dissociation for 60 s. The dissociation step occurred in the absence of CaCl_2_ and presence of 1 mM EDTA to ensure full dissociation between proteins (Fig. 6). We conducted the binding experiment over a dilution series of purified ecto-VLDLR in a 4 µL drop, so that we were able to calculate a *K*_D_ of the interaction by plotting the maximum response at each concentration, fitting the graph with a one site—specific binding, nonlinear regression (GraphPad). Each measurement was performed in triplicate, and the *K*_D_ of the interaction between the Fc-fused heterodimer and ecto-VLDLR was calculated to be 129 nM (Fig. 6, bottom). We performed this experiment for both the FLAG-WT homodimer-Fc and His6-K2467A homodimer-Fc, but not over a dilution series since the goal of the experiment was to demonstrate binding, or lack thereof, not calculate a *K*_D_. The interaction pattern was consistent with what was expected; the FLAG-WT homodimer-Fc and heterodimer-Fc constructs clearly interacted with ecto-VLDLR, while the His6-K2467A homodimer-Fc construct did not bind to the receptor (Fig. 7).

## Discussion

The production and purification of a heterodimeric protein starting with the cDNA that encodes a homodimeric protein is not trivial as it is reliant on low chance events. First, in stable cell line production, the same cell needs to acquire three independent plasmids (plasmid for chain A, chain B, and resistance against G418) in the appropriate proportion. Second, during selection of the clone, both A and B chains need to be integrated into similarly active genomic sites so that expression is equivalent. Third, their endoplasmic reticulum synthesis and oligomeric assembly should occur according to statistical probability into AA, BB and AB chains. Finally, accessibility and affinity of the FLAG and His6 tags should be similar so that during purification the AB heterodimer can be purified. Underscoring the low chance of the cumulative events having occurred, only 2 out of the roughly 50 tested clones that grew in the presence of the selective agent G418 expressed both the FLAG- and His6-tagged protein constructs (Fig. 1). While we could detect that both proteins were expressed in those two isolated clones, we could not interpret the degree to which each protein was expressed relative to each other given the inherent differences in antibody affinity and signal used to detect the presence of the two proteins. While this has potential implications on the heterodimer:homodimer ratio in the overall protein pool, the strategy of tandem affinity chromatography should result in a purified heterodimeric product that is 1:1 in its ratio of chain A:chain B.

Tandem affinity chromatography is also ideal for this purpose given that theoretically only a dimeric construct can be purified at the end. The IP, in which we pull down the construct targeting one chain (e.g., FLAG tagged chain), while probing for the other (e.g., His6 tagged chain) and vice versa, unequivocally demonstrates the heterodimeric structure of our purified product. Nonspecific binding and carry over as a result of insufficient washing are not supported as the homodimeric counterparts are not detected when subject to an identical procedure. The SEC traces support the claim that the heterodimer has a similar conformation in solution to both the WT and K2467A homodimers. The overlapping SEC profiles are indicative of the constructs having a similar size and shape, which is unsurprising given the minute differences in primary structure between the constructs (i.e., single amino acid mutation and different affinity tags).

The BLI experiment confirms the heterodimeric Reelin construct’s capacity to bind to the extracellular domain of VLDLR. Conducting the experiment over a dilution series, we calculated the *K*_D_ of the interaction between the heterodimer and ecto-VLDLR to be 129 nM (Fig. 6). Previous reports using surface plasmon resonance have calculated affinities between Reelin constructs and VLDLR/ApoER2 fragments to exist within the subnanomolar to nanomolar range (Hirai et al. 2017; Yasui et al. 2011, 2010). These data confirm that our heterodimer is functional as it binds to one of Reelin’s known receptors with a *K*_D_ consistent with previous examples of Reelin-receptor binding. Clear binding for the WT homodimer and absence of VLDLR interaction for the K2467A homodimer (Fig. 7), further confirm the integrity of our sample preparation and are in agreement with prior data that are foundational in the field of research regarding Reelin-receptor binding (Jossin et al. 2004; Yasui et al. 2010).

A parallel strategy we explored was to favor Fc heterodimerization by introducing “knob” and “hole” mutations, previously utilized to construct bispecific antibodies (Ridgway et al. 1996). Introducing distinct yet corresponding mutations on the Fc heavy chains to sterically favor their heterodimerization yields a pool of antibodies with a higher percentage of heterodimer versus homodimer. As our proteins are expressed as Fc fusion proteins, we applied this concept by mutating T366Y (knob) and Y407T (hole) on the Fc regions of construct A and construct B, respectively, to favor the formation of a heterodimeric construct AB. As a proof of principle, we used two Fc fusion proteins of measurably different molecular weights so that heterodimerization could be identified in SDS-PAGE. Co-transfection of the mutated Fc constructs to favor heterodimerization, followed by Protein A purification, did yield a heterodimeric pool of proteins (data not shown). Although viable, we did not pursue this strategy because we had successfully selected a stable cell line expressing both of our Reelin constructs as mentioned above.

A third strategy to obtain a heterodimer would be by reduction and re-oxidation of the disulfide bond responsible for Reelin’s dimeric conformation. However, we found that the formation of a heterodimer in oxidizing conditions after having reduced the proteins into their monomeric forms was nearly undetectable. Therefore, we refocused our efforts to relying on the cell’s biological machinery to drive the self-assembly of our heterodimeric target, with the aim to purify the fraction of interest out of the larger pool of overexpressed proteins.

The production and purification of this heterodimeric Reelin construct composed of a wild-type chain and mutated chain with an altered receptor binding site will be useful in dissecting the mechanism by which Reelin binds to its receptors and initiates signal transduction. This construct can be utilized in both biophysical and cell signaling assays to begin to answer why Reelin’s signaling capacity is dependent on its dimeric conformation. Analytical ultracentrifugation (AUC) is an obvious choice to study the biophysical aspects given the availability of this heterodimeric construct (Cole et al. 2008; Demeler et al. 2010). A direct comparison between heterodimer and its wild-type homodimeric counterpart in complex with the extracellular domains of VLDLR or ApoER2 could serve as a starting point for analyzing the stoichiometry of the binding event. Including the heterodimer among panels of Reelin constructs tested in cell signaling assays and measuring for markers of Reelin pathway activation such as Dab-1 phosphorylation or ribosomal protein S6 phosphorylation are themselves potential experiments to understand the importance of Reelin-receptor stoichiometry on signal transduction (Hiesberger et al. 1999; Lee et al. 2014).

## References

[CR1] Cole JL, Lary JW, Moody TP, Laue TM (2008). Analytical ultracentrifugation: sedimentation velocity and sedimentation equilibrium. Methods Cell Biol.

[CR2] D’Arcangelo G, Nakajima K, Miyata T, Ogawa M, Mikoshiba K, Curran T (1997). Reelin is a secreted glycoprotein recognized by the CR-50 monoclonal antibody. J Neurosci.

[CR3] D’Arcangelo G, Homayouni R, Keshvara L, Rice DS, Sheldon M, Curran T (1999). Reelin is a ligand for lipoprotein receptors. Neuron.

[CR4] Demeler B, Brookes E, Wang R, Schirf V, Kim CA (2010). Characterization of reversible associations by sedimentation velocity with UltraScan. Macromol Biosci.

[CR5] Hiesberger T, Trommsdorff M, Howell BW, Goffinet A, Mumby MC, Cooper JA, Herz J (1999). Direct binding of Reelin to VLDL receptor and ApoE receptor 2 induces tyrosine phosphorylation of disabled-1 and modulates tau phosphorylation. Neuron.

[CR6] Hirai H, Yasui N, Yamashita K, Tabata S, Yamamoto M, Takagi J, Nogi T (2017). Structural basis for ligand capture and release by the endocytic receptor ApoER2. EMBO Rep.

[CR7] Jossin Y, Ignatova N, Hiesberger T, Herz J, Lambert de Rouvroit C, Goffinet AM (2004). The central fragment of Reelin, generated by proteolytic processing in vivo, is critical to its function during cortical plate development. J Neurosci.

[CR8] Kubo K, Mikoshiba K, Nakajima K (2002). Secreted Reelin molecules form homodimers. Neurosci Res.

[CR9] Lee GH, D’Arcangelo G (2016). New insights into reelin-mediated signaling pathways. Front Cell Neurosc.

[CR10] Lee GH, Chhangawala Z, von Daake S, Savas JN, Yates JR, Comoletti D, D'Arcangelo G (2014). Reelin induces Erk1/2 signaling in cortical neurons through a non-canonical pathway. J Biol Chem.

[CR11] Ridgway JB, Presta LG, Carter P (1996). ‘Knobs-into-holes’ engineering of antibody CH3 domains for heavy chain heterodimerization. Protein Eng.

[CR12] Strasser V, Fasching D, Hauser C, Mayer H, Bock HH, Hiesberger T, Herz J, Weeber EJ, Sweatt JD, Pramatarova A (2004). Receptor clustering is involved in Reelin signaling. Mol Cell Biol.

[CR13] Yasui N, Nogi T, Takagi J (2010). Structural basis for specific recognition of reelin by its receptors. Structure.

[CR14] Yasui N, Kitago Y, Beppu A, Kohno T, Morishita S, Gomi H, Nagae M, Hattori M, Takagi J (2011). Functional importance of covalent homodimer of reelin protein linked via its central region. J Biol Chem.

